# Cholestasis impairs hepatic lipid storage via AMPK and CREB signaling in hepatitis B virus surface protein transgenic mice

**DOI:** 10.1038/s41374-020-0457-9

**Published:** 2020-07-01

**Authors:** Karuna Irungbam, Martin Roderfeld, Hannah Glimm, Felix Hempel, Franziska Schneider, Laura Hehr, Dieter Glebe, Yuri Churin, Gertrud Morlock, Imanuel Yüce, Elke Roeb

**Affiliations:** 1 Department of Gastroenterology, Justus Liebig University Giessen, Giessen, Germany; 2 Institute of Medical Virology, National Reference Centre for Hepatitis B Viruses and Hepatitis D Viruses, Justus Liebig University, Giessen, Germany; 3 Institute of Nutritional Science, Chair of Food Science, and TransMIT Center for Effect-Directed Analysis, Justus Liebig University Giessen, Giessen, Germany

**Keywords:** Non-alcoholic fatty liver disease, Experimental models of disease

## Abstract

Clinical studies demonstrated that nonalcoholic steatohepatitis is associated with liver-related outcomes in chronic hepatitis B. Furthermore, primary biliary fibrosis and biliary atresia occurred in patients with HBV infection. Interestingly, hepatitis B virus surface protein (HBs) transgenic mice spontaneously develop hepatic steatosis. Our aim is to investigate the effect of *Abcb4* knockout-induced cholestasis on liver steatosis in HBs transgenic mice. Hybrids of HBs transgenic and Abcb4^−/−^ mice were bred on the BALB/c genetic background. Lipid synthesis, storage, and catabolism as well as proteins and genes that control lipid metabolism were analyzed using HPTLC, qPCR, western blot, electrophoretic mobility shift assay (EMSA), lipid staining, and immunohistochemistry. Hepatic neutral lipid depots were increased in HBs transgenic mice and remarkably reduced in Abcb4^−/−^ and HBs/Abcb4^−/−^ mice. Similarly, HPTLC-based quantification analyses of total hepatic lipid extracts revealed a significant reduction in the amount of triacylglycerols (TAG), while the amount of free fatty acids (FFA) was increased in Abcb4^−/−^ and HBs/Abcb4^−/−^ in comparison to wild-type and HBs mice. PLIN2, a lipid droplet-associated protein, was less expressed in Abcb4^−/−^ and HBs/Abcb4^−/−^. The expression of genes-encoding proteins involved in TAG synthesis and de novo lipogenesis (*Agpat1*, *Gpat1*, *Mgat1*, *Dgat1*, *Dgat2*, *Fasn*, *Hmgcs1*, *Acc1*, *Srebp1-c*, and *Pparγ*) was suppressed, and AMPK and CREB were activated in Abcb4^−/−^ and HBs/Abcb4^−/−^ compared to wild-type and HBs mice. Simulating cholestatic conditions in cell culture resulted in AMPK and CREB activation while FASN and PLIN2 were reduced. A concurrent inhibition of AMPK signaling revealed normal expression level of FASN and PLIN2, suggesting that activation of AMPK–CREB signaling regulates hepatic lipid metabolism, i.e. synthesis and storage, under cholestatic condition. In conclusions, in vivo and mechanistic in vitro data suggest that cholestasis reduces hepatic lipid storage via AMPK and CREB signaling. The results of the current study could be the basis for novel therapeutic strategies as NASH is a crucial factor that can aggravate chronic liver diseases.

## Abstract (web summary)

The authors demonstrate that cholestasis impairs hepatic lipid storage via AMP-activated protein kinase (AMPK) and CREB signaling in hepatitis B virus surface protein transgenic mice. The pharmacological modulation of AMPK and CREB signaling might be a promising therapeutic concept for the treatment of fatty liver diseases.

## Introduction

The injury of bile ducts is a hallmark of chronic cholestatic liver disorders of multifactorial origin leading to hepatic accumulation of bile acids (BA) and subsequent liver tissue damage [1, 2]. Bile production is a complex process involving hepatocytes, cholangiocytes, and a number of different bile acid transporters that coordinate bile formation [1]. ATP-binding cassette subfamily B member 4 (ABCB4) is a phospholipid translocator at the canalicular membrane of the hepatocyte, which “flops” phosphatidylcholine into the bile. Phosphatidylcholines are essential for the formation of bile acid-containing micelles, a crucial process in protecting cholangiocyte membranes from being exposed to high concentrations of free cell-toxic BA [2]. Dysfunction or deficiency of this transporter can cause liver diseases such as progressive familial intrahepatic cholestasis type 3, low phospholipid-associated cholelithiasis, intrahepatic cholestasis of pregnancy, drug-induced liver injury, and chronic cholangiopathy with biliary fibrosis and cirrhosis [2]. Similar to human cholestatic liver diseases, *Abcb4* knockout mice develop liver fibrosis, multiple derangements of lipid metabolism, including alterations in cholesterol and phospholipid metabolism [3, 4]. Chronic viral hepatitis (CVH) implies liver damage causing liver fibrosis and subsequent hepatocellular carcinoma formation [5]. It has been shown recently in a large combined tertiary center cohort that patients with concomitant nonalcoholic steatohepatitis (NASH) and chronic hepatitis B infection had poorer clinical outcomes [6]. Interestingly, the transgenic mice overexpressing HBV surface proteins without viral infection [7] that were used in the current study, developed hepatic steatosis [8]. Apart from steatosis, further histopathological changes have been reported in the same transgenic model including inflammation, regenerative hyperplasia, endoplasmatic reticulum stress, and associated unfolded protein response [9–11].

Characteristics of the metabolic syndrome like obesity, hypertension, and lipometabolic disorders are associated with the presence of NASH in patients with chronic hepatitis B [12]. Moreover, concurrent NASH drives a “second hit” to the liver in patients with chronic HBV infection [6] and even noninvasive score models for the prediction of NASH in patients with chronic hepatitis B and superimposed nonalcoholic fatty liver diseases (NAFLD) have been suggested [13]. NAFLD depicts the leading cause of liver diseases in the Western world [14]. Apart from HBV, also other risk factors like malnutrition, gut microbes, and drugs can aggravate NASH [15–17].

Adenosine monophosphate-activated kinase (AMPK) is believed to act as a key master switch that modulates lipid metabolism by directly phosphorylating proteins or modulating gene transcription in specific tissues such as liver, fat, and muscle [18, 19]. SREBP1c, P-ACC1, FASN, SCD1, HMGCS1, GPAT1, etc are involved either in synthesis, oxidation, or lipolysis, which are modulated directly through phosphorylation or modulation at gene transcription level by activated AMPK [19]. In addition, activation of AMPK also activates the expression of CREB [20]. The most abundant proteins associated with lipid droplets (LDs) belong to the perilipin (PLIN) protein family [21]. PLIN proteins (PLIN1–PLIN5) are important regulators of cellular lipid metabolism, directly controlling how and when cells and tissues store, mobilize, and utilize lipids. Previous studies reported on the activated AMPK-mediated phosphorylation of LD associated protein PLIN2 and subsequent triggering of lipolysis [22], suggesting an interplay between AMPK and PLIN2 in maintaining intracellular triacylglycerols (TAG) storage.

The dysregulation of hepatic and systemic lipid metabolism in Abcb4^−/−^ mice has been described before [3]. Interestingly, the authors reported that liver injury was critically linked to impaired lipid homeostasis, which was ameliorated by norUDCA treatment and additionally by dietary intervention in Abcb4^−/−^ mice under HFD. Similarly, we previously reported that hepatitis B virus surface proteins could accelerate cholestatic liver injury and tumor progression in Abcb4^−/−^ mice reflecting a model of simultaneous liver damage with enhanced carcinogenesis [23]. Based on these findings, the present study was designed to investigate the effect of *Abcb4* knockout-induced cholestasis on lipid metabolism in HBs transgenic mice.

## Material and methods

### Animal experiments

BALB/c-Abcb4 mice (C.FVB (129P2)-Abcb4^tm1Bor^ herein called Abcb4^−/−^ mice) were bred and housed as described previously [24]. Characterization of Abcb4^−/−^ genotype, sample collection, and routine analysis has been described elsewhere [25]. Generation and characteristics of transgenic lineages Tg (Alb1HBV) (C57BL/6J-Tg (Alb1HBV) 44Bri/J) have been described [10]. These mice were crossed to BALB/cJ background (C.B6J-Tg (Alb1HBV) 44Bri herein called HBs mice) for nine generations. HBs mice were crossed with Abcb4^−/−^ mice, resulting in the F2 generation BALB/c-Abcb4/Alb1HBV hybrid mice (C.Cg-Tg (Alb1HBV) 44Bri-Abcb4^tm1Bor^ herein called HBs/Abcb4^−/−^ mice). All mice were housed in a pathogen-free environment under a constant 12-h light–dark cycle at 22 °C temperature and 50% humidity. The mice were fed standard chow (Altromin, Lage, Germany) and water ad libitum.

Mice were sacrificed at the age of 12–19 weeks (*n* = 4–5 per age and sex). Livers were collected and underwent morphological diagnosis. Remaining samples were preserved for analyses as indicated. Serum samples were stored at −80 °C until analysis of serum TAGs and cholesterol using the Reflotron plus Analyzer (Roche, Mannheim, Germany). This study was carried out in strict accordance to the recommendations laid in the guide for the care and use of laboratory animals of the German law of animal welfare. All experiments were approved by the committee on the ethics of animal experiments of the Regierungspraesidium Giessen, Giessen, Germany (permit number: V54-19c 2015c GI20/10 Nr. A36/2011, Nr. A5/2012, and Nr. 52_2011).

### Western blot

Liver and cell lysates were prepared in 1× laemmli buffer and boiled at 95 °C for 10 min, and then briefly centrifuged for 5 min. After SDS-PAGE, proteins were transferred to a nitrocellulose membrane following the standard protocol [26]. Protein detection was performed using specific antibodies against AMPK (Genetex: #GTX50863-100), Phospho-AMPKα (Thr172) (CST: #2531)), Perilipin2/PLIN2 (Proteintech: #15294-1-AP, CIDEC: #198204), Phospho-CREB (Ser133) (87G3) rabbit mAb (CST: #9198), CREB (CST: #9104), and GAPDH (Proteintech: 60004-1-Ig). The proteins were visualized using peroxidase-conjugated secondary antibodies and chemoluminescent reagent developed on Intas ECL chemostar (Göttingen, Germany). Alternatively, visualization was performed using alkaline phosphatase conjugated secondary antibodies with soluble 5-bromo-4-chloro-3-indolyl phosphate and nitroblue tetrazolium.

### Immunohistochemistry

Immunohistochemistry (IHC) was performed using Impress Peroxidase/Alkaline Detection Reagents (Vector Laboratories) and antibodies specific for PLIN2 (Proteintech: #15294-1-AP), CIDEC (Novus: NBP1-76950), lysosomal acid lipase (LAL) (Novus: NBPI-54155SS), Lipoprotein lipase (LPL) (Proteintech: #21133-1-AP), Phospho-CREB (Ser133) (CST: #9198), DGAT1 (Proteintech: #11561-1-AP), or MGAT1 (Santa Cruz: #sc376079). Color reaction was developed with VECTOR VIP Peroxidase Substrate Kit or DAB Peroxidase Substrate Kit (Vector Laboratories, USA) or HighDef® red IHC AP chromogen (Enzo, USA).

### Sample preparation and real-time PCR

RNA isolation was conducted according to the manufacturer’s protocol using the Direct-zol RNA extraction kit (Zymo research). cDNA synthesis was produced using the High capacity cDNA synthesis kit (Thermo, 4374966). Real-time qPCR was performed according to the protocol described before [25]. Primers were ordered from Microsynth (Switzerland). qPCR data were analyzed using the ΔΔC_T_ method [27]. Sequences and properties of primers are listed in Table 1.

**Table 1:** qRT-PCR primer list.

Gene	Forward	Reverse	Primer bank ID
*Mtt*	5′-CTC TTG GCA GTG CTT TTT CTC T-3′	5′-GAG CTT GTA TAG CCG CTC ATT-3′	6678960A1
*Plin2*	5′-GAC CTT GTG TCC TCC GCT TAT-3′	5′-CAA CCG CAA TTT GTG GCT C-3′	31982516a1
*Gapdh*	5′-GGC TGT ATT CCC CTC CAT CG-3′	5′-CCA GTT GGT AAC AAT GCC ATG T -3′	–
*Fasn*	5′-GGA GGT GGT GAT AGC CGG TAT-3´	5′-TGG GTA ATC CAT AGA GCC CAG-3′	30911099a1
*Scd-1*	5′-TTC TTG CGA TAC ACT CTG GTG C-3′	5′-CGG GAT TGA ATG TTC TTG TCG T-3′	31543675a1
*Srebp-1c*	5′-GATGTGCGAACTGGACACAG-3′	5′-CAT AGG GGG CGT CAA ACA G-3′	27753981a1
*Lpl*	5′-GGGAGTTTGGCTCCAGAGTTT-3′	5′-TGT GTC TTC AGG GGT CCT TAG-3′	6678710a1
*Dgat1*	5′-TCC GTC CAG GGT GGT AGT G-3′	5′-TGA ACA AAG AAT CTT GCA GAC GA-3′	6753632a1
*Dgat2*	5′-GCG CTA CTT CCG AGA CTA CTT-3′	5′-GGG CCT TAT GCC AGG AAA CT-3′	16975490a1
*Acot1*	5′-ATA CCC CCT GTG ACT AC CTG-3′	5′-CAA ACA CTC ACT ACC CAA CTG-3′	6753550a1
*Cd36*	5′-ATG GGC GT GAT CGG AAC TG-3′	5′-GTC TTC CCA ATA AGC ATG TCT-3′	31982474a1
*Pparγ*	5′-AGA GCC CCA TCT GTC CTC TC-3′	5′-ACT GGT AGT CTG CAA AAC CAA-3′	18875426a1
*Apoe*	5′-CTG ACA GGA TGC CTA GCC G-3′	5′-CGC AGG TAA TCC CAG AAG C-3′	6753102a1
*Vldlr*	5′-GGC AGC AGG CAA TGC AAT G-3′	5′-GGG CTC GTC ACT CCA GTC T-3′	15489005a1
*Pctp*	5′-TTC TCG GAC GAG CAG TCC C-3′	5′-CCG GTA GAT GGT TAT GCC TGA G-3′	6679235a1
*Apoe*	5′-CTG ACA GGA TGC CTA GCC G-3′	5′-CGC AGG TAA TCC CAG AAG C-3′	6753102a1
*Mgl*	5′-ACC ATG CTG TGA TGC TCT CTG-3′	5′-CAA ACG CCT CGG GGA TAA CC-3′	12840263a1
*Hsl*	5′-CCTGGCAAGCCTCATCGTC-3′	5′-AGC AGC CCG GCT AGT AGT AG-3′	27923943a1
*Gpat*	5′-CAGCCAGGTTCTACGCCAAG-3′	5′-TGA TGC TCA TGT TAT CCA CGG T-3′	23956162a1
*Agpat1*	5′-TAAGATGGCCTTCTACAACGGC-3′	5′-CCA TAC AGG TAT TTG ACG TGG AG-3′	26352009a1
*β-actin*	5′-CAG CTT CTT TGC AGC TCC TT-3′	5′-AGT CCT TCT GAC CCA TTC CC-3′	500747a1

### Oil red O staining

Oil red O staining was performed as described previously [28]. Staining was assessed by bright-field microscopy.

### High performance thin layer chromatography (HPTLC)

The liver tissue extracts were prepared as described [29] with slight modifications. Briefly, 20 mg of frozen liver tissue were weighed and subsequently homogenized with 1.0 mL n-hexane/2-propanol 3:2 (*V*/*V*) for 1 h. This suspension was centrifuged at 4 °C at 10,000 × *g* for 10 min. The supernatant was transferred to a vial, dried under nitrogen gas and the residue was resuspended in 100 µL of chloroform/methanol 2:1 (*V*/*V*). For application on the HPTLC plate, this stock solution was further diluted 1:4 in chloroform/methanol 1:1 (*V*/*V*). Individual lipid stock solutions (25 mg/mL) were mixed to obtain a lipid standard mixture of 300 ng/µL each in chloroform/methanol 1:1 (*V*/*V*). The sample and standard solutions were sprayed with the Automatic TLC Sampler 4 (ATS4, CAMAG, Muttenz, Switzerland) as 8-mm bands allowing up to 21 tracks to be applied on one HPTLC plate of 20 × 10 cm (distance from lower edge 8 mm, distance from left edge 14.5 mm, automatic distance between bands). For calibration, 0.3, 6, 12, and 21 µg of the standard mixture solution were sprayed on the HPTLC plate (300–2100 ng/band) along with 2.5 µL liver extract samples. After drying of the start zones for 0.5 min (hair dryer), development was performed according to [30] on an HPTLC plate silica gel 60 F_254_-MS-grade (Merck, Darmstadt, Germany; preheated to 110 °C for 15 min) with n-hexane/diethyl ether/acetic acid (8:2:0.4, *V*/*V*/*V*) in the Twin-Through chamber (CAMAG, with filter paper) pre-saturated for 20 min. The developing distance was 65 mm (from the lower edge of the plate). After plate drying for 2 min, detection was performed by immersion into primuline reagent (100 mg primuline in 200 mL acetone/water 4:1, *V*/*V*) at an immersion speed of 3 cm/s and an immersion time of 1 s using the TLC Chromatogram Immersion Device III (CAMAG). After plate drying for 2 min, the fluorescence measurement was performed at 366/>400 nm using the TLC scanner 4 (CAMAG, mercury lamp, measurement slit 6.0 mm × 0.2 mm, scanning speed 20 mm/s, optical filter K400). The chromatogram was documented at UV 366 nm via the TLC Visualizer (CAMAG). All instrumentation and data processing were operated with the winCATS software (CAMAG, version 1.4.6.2002).

For qualitative visualization, HPTLC plates were derivatized using sulfuric acid-anisaldehyde reagent (201 mL sulfuric acid/acetic acid/methanol/anisaldehyde, 1:2:17:0.1, *V/V/V/V*). The plate was immersed into the derivatization reagent at a speed of 3.5 cm/s and a time of 1 s, followed by heating at 110 °C for 9 min. The chromatograms were subsequently documented at white light illumination.

The selected zones were online transferred with methanol (flow rate 0.1 mL/min) using the TLC-MS Interface 2 (equipped with an elution head, 4 × 2 mm, CAMAG) coupled to the Q Exactive Plus Hybrid Quadrupole-Orbitrap Mass Spectrometer (Thermo Fisher Scientific). Between the interface and the mass spectrometer, a filter frit (Upchurch Scientific A-356 and PEEK-Frit Blue UPA-703, Techlab, Erkerode, Germany) was installed to prevent the mass spectrometer from particles. As ion source was used atmospheric pressure chemical ionization. The spectrometer was operated and spectra were recorded with Xcalibur 3.0.63 software (Thermo Fisher Scientific). High-resolution mass spectra were measured as full scan at a resolution of 280,000 in the range of *m/z* 100–1000.

### Electrophoretic mobility shift assay (EMSA)

The target oligo’s probe sequence for CREB consensus binding site was provided by Santa Cruz Biotechnology, Inc. (sc-2504). CREB consensus sequences (F) 5′-AGA GAT TGC CTG ACG TCA GAG AGC TG-3′(R) 5′-CTA GCT CTC TGA CGT CAG GCA ATC TCT-3′. PPAR consensus binding sequences: (F) 5′-CAA AAC TG GTC AAA GGT CA-3′, (R) 5′-TGA CCT TTG ACC TG TTT TG-3′. The 5′ ends of all single-stranded probes were biotin labeled. Nonbiotin-labeled probes were also used as competitor probe (Microsynth, Germany).

Nuclear proteins extraction from liver samples was performed using NE-PER™ Nuclear and Cytoplasmic Extraction Reagents (Thermo scientific, cat no. 78833). For the EMSA reaction, 2 μg of the nuclear protein was incubated with reaction reagents as per the protocol given in Light shift Chemiluminescent EMSA Kit (Thermo Scientific, cat no 20148). After a 10 min equilibration to 25 °C, biotin labeled (hot probe) and excess of nonbiotin probes (cold probe) were added and further incubated for 20–30 min at room temperature. After hybridization, the complexes were resolved by electrophoresis on 5% nondenaturing polyacrylamide gels in 0.5× TBE (Tris-borate-EDTA) buffer at 10 V/cm for 1 h. The gel was subsequently transferred to a positively charged nylon membrane using a semidry transfer cell. DNA was detected using Light shift Chemiluminescent EMSA Kit (cat no 20148, Thermo Scientific). Images were processed by Intas Imager (ECL chemostar, Intas, Germany).

### Lipase activity assay

Liver samples were weighed and lysates prepared in PBS plus protease inhibitor in cold condition as per protocol (Cayman cat no. 700640).

### Free fatty acid (FFA) quantification

Liver samples were weighed and lysates were prepared as per protocol for FFA quantification (Promokine, cat no. PK-CA577-K612).

### TAG colorimetric assay

Liver samples were weighed and lysates prepared as recommended by the manufacturer (Cayman, cat no. 10010303). The assay was initiated with the enzymatic hydrolysis of the TAGs by lipase to produce glycerol and FFAs. The glycerol released was subsequently measured by a coupled enzymatic reaction system with a colorimetric readout at 540 nm.

### HepG2 cell culture

HepG2 cell lines were obtained from CLS services Germany. HepG2 cells were grown at 37 °C in an atmosphere of 5% CO_2_, 95% air in cell culture dish using 10 mL of DMEM–F-12 medium with 10% fetal calf serum, 1% penicillin, 1% streptomycin, and 1% fungizone. Cells were plated at a split ratio of 1:4. The preconfluent cells were left either untreated (control cells) or pretreated with oleic acid (BSA conjugated oleic acid, Sigma cat no. O3008-5ML), at a concentration of 250 μM for 12 h. Subsequent treatment with BA (Sigma-Aldrich, cat no. 48305-50G-F) was performed in serum free DMEM–F-12 medium. Similarly, for inhibitor assays, the cells were pretreated with Dorsomorphin (AMPK inhibitor, Merck, P5499, 10 µM) for 1 h prior to treatment with the BA. The plasmid pCH9/200LMS is a replication-defective variant of plasmid pCH9/3091 [31] and encodes the HBV surface proteins under their natural promoters. HepG2 cells with 80% confluence were transfected with and without the plasmid pCH9/200LMS for 48 h, followed by treatment with oleic acid for 12 h. After that treated with bile acid in serum free DMEM for the next 24 h.

### Statistics

The results are presented as means ± SEM. One-way ANOVA followed by Tukey’s multiple comparisons test was performed using GraphPad Prism version 7.00 for Windows, GraphPad Software, La Jolla, CA, USA, and controlled with SPSS26.0, IBM, Ehningen, Germany.

## Results

### *Abcb4* knockout reduced hepatic lipid accumulation in HBs transgenic mice

**Fig. 1:**
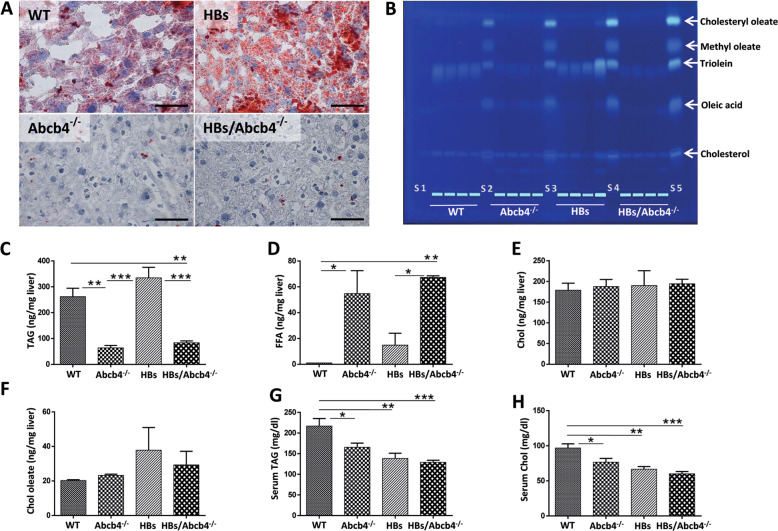
Reduced lipid accumulation in HBs/Abcb4^−/−^ mice. **a** Representative Oil red O staining demonstrated decreased accumulation of neutral lipids in Abcb4^−/−^ and HBs/Abcb4^−/−^ in comparison to WT and HBs mice (original image magnification ×1000, bar 50 µm). **b** Representative HPTLC-FLD chromatogram at 366 nm of the nonpolar lipids after AMD2 separation and derivatization with the primuline reagent. **c**–**f** Mean content of TAGs (triolein equivalents), cholesterol, FFAs (oleic acid equivalents), and cholesteryl oleate in the liver extracts determined after densitometric fluorescence measurement at 366/>400 nm. The results represent the mean ± SEM from at least two independent HPTLC plates with four independent liver extracts (*n* = 4, 2♂ + 2♀, age 16–19 weeks) in each group. **g**, **h** Serum TAGs and cholesterol estimation using Reflotron kit with *n* = 10 (5♂ + 5♀, age 16–19 weeks) in each per group. *P < 0.05, **P < 0.01, ***P < 0.001.

We investigated the effects of Abcb4^−/−^-induced cholestasis in two distinct models, Abcb4-deficient mice (Abcb4^−/−^) and HBs overexpressing mice as well as chimera of both, knockout and transgene, on BALB/c genetic background (HBs, HBs/Abcb4^−/−^). Oil red O staining revealed a remarkable reduction of neutral lipid depots in the hepatocytes of both, Abcb4^−/−^ and HBs/Abcb4^−/−^, mice in comparison to wild-type and HBs transgenic mice, respectively, (Fig. 1a).

An HPTLC method with fluorescence detection after derivatization with the primuline reagent (HPTLC-FLD) allowed the separation and detection of five different lipid species (cholesterol, FFAs with oleic acid as reference, TAG, methyl oleate, and cholesteryl oleate) in liver extract samples. The lipid fractions were assigned using a lipid standard mixture (Fig. 1b). These preliminary assignments were confirmed by high-resolution mass spectrometry (Supplementary Fig. 1a–d). The HPTLC-FLD chromatogram and its quantitative analysis revealed a decrease in hepatic TAGs in Abcb4^−/−^ mice as compared to wild type (Fig. 1c). HBs mice presented with higher hepatic TAG levels than Abcb4^−/−^ mice, which were reduced in HBs/Abcb4^−/−^ (Fig. 1c). The hepatic levels of FFA, on the other hand, were significantly increased in Abcb4-deficient mice and HBs/Abcb4^−/−^ in comparison to wild-type and HBs mice, respectively (Fig. 1d). The amount of hepatic cholesterol and cholesteryl oleate was not altered among groups (Fig. 1e, f). Alternative HPTLC assessments of TAGs and FFAs strengthened this outcome and displayed comparable results (Supplementary Fig. 1a, b).

Serum levels of TAGs and cholesterol were reduced in all groups in comparison to WT but not altered in HBs/Abcb4^−/−^ mice compared to HBs and Abcb4^−/−^ mice, respectively, (Fig. 1g, h). Taken together, TAGs were reduced in liver but not in the serum of HBs/Abcb4^−/−^ compared to HBs mice.

### *Abcb4* knockout altered hepatic lipid metabolism in HBs/Abcb4^−/−^ mice

The reduction of TAGs in hepatocytes of HBs/Abcb4^−/−^ mice in comparison to HBs mice prompted us to investigate the genes involved in de novo lipogenesis, lipid uptake, and transport.

**Fig. 2:**
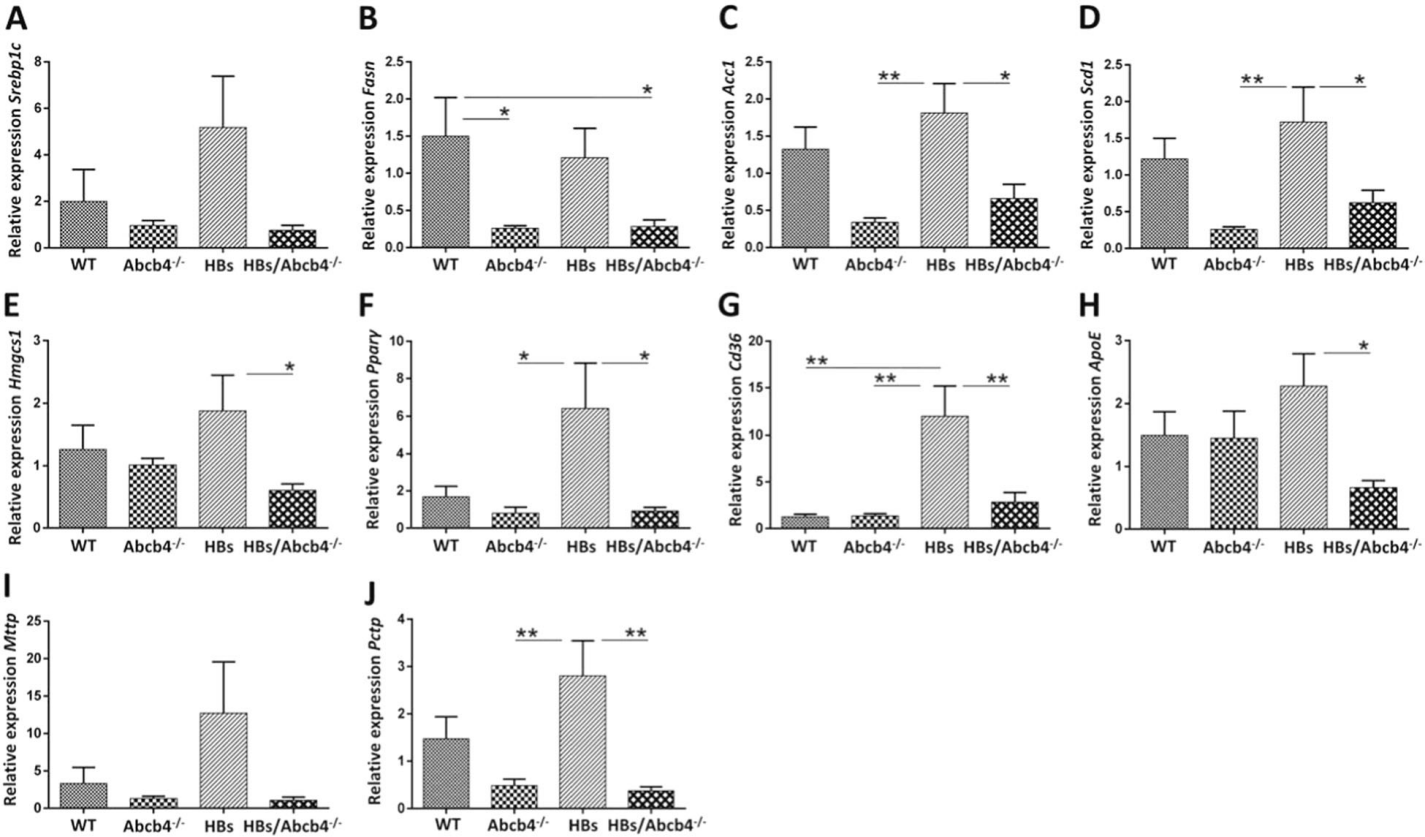
Alteration of hepatic lipid metabolism in HBs/Abcb4^−/−^ mice. **a**–**j** Relative mRNA expression of major genes involved in de novo lipogenesis, SREBP-1c, FASN, ACC1, Scd1, Hmgcs1, PPARγ, CD36, ApoE, MTTP, and PCTP in livers of four different groups, normalized against β-actin gene. Housekeeping genes and primers used are listed in Table 1. Total RNA was isolated, cDNA synthesized and relative quantitation was performed in applied biosystem step one real-time PCR system (Bio-Rad). Individual fold changes relative to WT were calculated and presented as mean ± SEM, *n* = 7–10 (3–5♂ + 4–5♀, age 16–19 weeks) in each group performed in duplicates. *P < 0.05, **P < 0.01.

Although *Srebp-1c* appeared increased in HBs and reduced in HBs/Abcb4^−/−^ by trend, statistical significance was not reached and the expression of *Srebp-1c* was not altered in Abcb4^−/−^, HBs, and HBs/Abcb4^−/−^ mice in comparison to wild type (Fig. 2a).

Fatty acid synthase (*Fasn*) gene expression was reduced in Abcb4^−/−^ and HBs/Abcb4^−/−^ compared to wild type (Fig. 2b). *Acc1* and *Scd1* were reduced in Abcb4^−/−^ and HBs/Abcb4^−/−^ compared to HBs mice (Fig. 2c, d). *Hmgcs1*was reduced in HBs/Abcb4^−/−^ compared to HBs mice (Fig. 2e). The transcription factor *Pparγ* was downregulated in Abcb4^−/−^ and HBs/Abcb4^−/−^ mice compared to HBs (Fig. 2f).

Furthermore, *Cd36* (fatty acid translocase) was induced in HBs compared to WT and reduced in Abcb4^−/−^ and HBs/Abcb4^−/−^ mice compared to HBs (Fig. 2g). *ApoE* was reduced in HBs/Abcb4^−/−^ mice compared to HBs (Fig. 2h). Although *Mttp* appeared increased in HBs and reduced in HBs/Abcb4^−/−^ by trend, statistical significance was not reached and *Mttp* (Microsomal TAG transfer protein) was not altered (Fig. 2i). Phosphatidylcholine transfer protein (*Pctp*), responsible for transfer of phosphatidylcholine, was reduced in Abcb4^−/−^ and HBs/Abcb4^−/−^ compared to HBs (Fig. 2j).

Taken together, our results indicate that *Abcb4* knockout suppressed the expression of genes involved in de novo lipogenesis as well as transcription factors and genes involved in lipid transport in HBs/Abcb4^−/−^ mice.

### *Abcb4* knockout reduced hepatic lipid droplet-associated protein PLIN2, TAG synthesis, and TAG storage in HBs mice

**Fig. 3:**
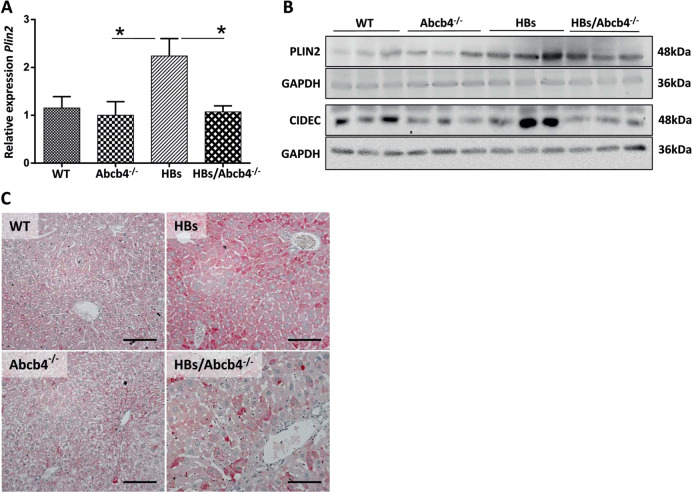
Reduced expression of LDs associated protein, PLIN2 in HBs/Abcb4^−/−^ mice. **a** Graph representing hepatic mRNA expression of PLIN2 gene relative to gene expression in WT of four different mice groups, normalized against GAPDH gene. *n* = 7–10 (3–5♂ + 4–5♀, age 16–19 weeks), *P < 0.05. **b** Representative western blot analysis demonstrated reduced levels of PLIN2 and CIDEC in liver lysates of Abcb4^−/−^ and HBs/Abcb4^−/−^ in comparison to WT and HBs transgenic in male mice. Equal loading was confirmed by GAPDH analysis. **c** Representative immunohistochemical analysis of PLIN2 depicts decreased expression in Abcb4^−/−^ and HBs/Abcb4^−/−^ in comparison to WT and HBs transgenic male mice. Original image magnification ×200, bar 100 µm.

Among PLIN proteins, PLIN2 is the constitutive and ubiquitously expressed protein that has been used as a marker for LDs, correlating with the amount of LDs and TAG storage [32]. We therefore investigated the effect of an *Abcb4* knockout on the PLIN2 expression as an additional marker for intracellular lipid storage. Real-time PCR, western blot, and IHC revealed a reduced expression of PLIN2 in HBs/Abcb4^−/−^ compared to HBs transgenic mice (Fig. 3a–c). Western blotting analyses suggested a similar reduction of hepatic PLIN2 in female mice (Supplementary Fig. 2a, b). Plin2 is stained red. The protein expression of PLIN3 and PLIN5, however, was not regulated (data not shown). CIDEC, another LD-associated protein, showed a remarkable reduction in protein expression in Abcb4^−/−^ and HBs/Abcb4^−/−^ mice compared to wild-type and HBs transgenic mice (Fig. 3b). In conclusion, the reduction of PLIN2 might be considered as an indicator of reduced TAG storage in consequence of the *Abcb4* knockout.

**Fig. 4:**
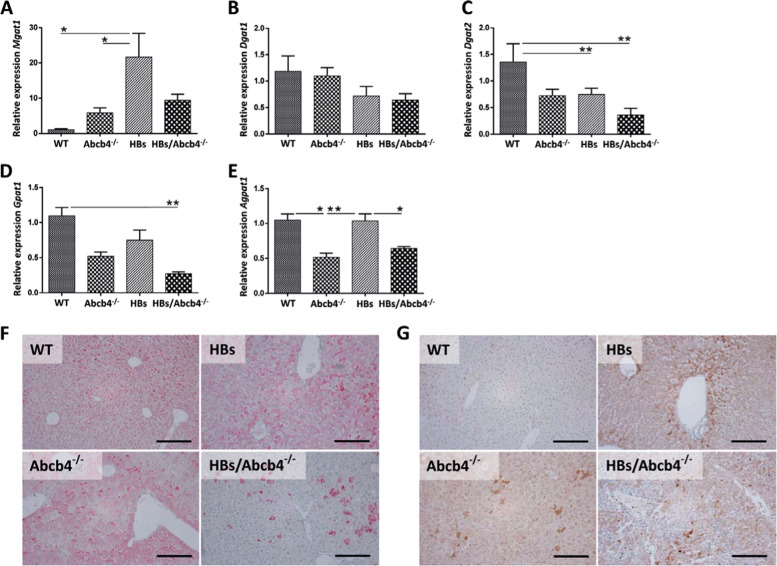
Reduced TAG synthesis and storage in HBs/Abcb4^−/−^ mice. **a**–**e** Graph representing mRNA expression of major genes involved in triacylglycerol synthesis; MGAT1, DGAT1, DGAT2, GPAT1, and AGPAT1 in the liver of four different groups, relative to WT and normalized against β-actin gene. The primers used are listed in Table 1. Total RNA was isolated, cDNA synthesized and relative quantitation was performed in applied biosystem step one real-time PCR system. The results are presented as mean ± SEM, *n* = 7–10 (3–5♂ + 4–5♀, age 16–19 weeks) in each group performed in duplicates. *P < 0.05, **P < 0.01. **f** Representative immunohistochemical staining using specific antibodies against DGAT1 was performed with male mice (original image magnification ×200, bar 200 µm). **g** Representative immunohistochemical staining using specific antibodies against MGAT1 in male mice. Original image magnification ×200, bar 200 µm.

Thus, we further investigated genes mainly involved in TAG synthesis pathways. Transcription of *Mgat1*was highly upregulated by HBs (Fig. 4a). Diacylglycerol acyltransferase 1 (DGAT1) and DGAT2 both catalyze the final committed step of TAG synthesis. At transcriptional level, Dgat2 was downregulated in HBs and HBs/Abcb4^−/−^ as compared to WT, while Dgat1 showed no significant regulation (Fig. 4b, c). Similarly, acyl-CoA: glycerol-3-phosphate acyltransferase (GPAT) and acyl-CoA: 1-acyl-glycerol-3-phosphate acyltransferase (AGPAT) are involved in the de novo synthesis of TAGs in the glycerol-3-phosphate pathway. Our results displayed a downregulation of *Gpat1* in HBs/Abcb4^−/−^ mice in comparison to WT but only in tendency in comparison to HBs (*P* = 0.081). *Agpat1* was reduced at transcriptional level in both, Abcb4^−/−^ and HBs/Abcb4^−/−^ mice, compared to wild-type and HBs mice (Fig. 4d, e). Taken together our findings suggest that the reduction of TAG synthesis is associated with the reduction of LDs and LD-associated proteins in Abcb4-deficient HBs mice. DGAT1 protein expression, however, showed a remarkable reduction in HBs/Abcb4^−/−^ in comparison to Abcb4^−/−^ and HBs mice (Fig. 4f). The protein expression of MGAT1, which synthesizes DAGs by catalyzing the acylation of monoacylglycerols (MAGs), showed a slight reduction in HBs/Abcb4^−/−^ compared to HBs (Fig. 4g).

### *Abcb4* knockout enhanced lipolysis in HBs/Abcb4^−/−^ mice

**Fig. 5:**
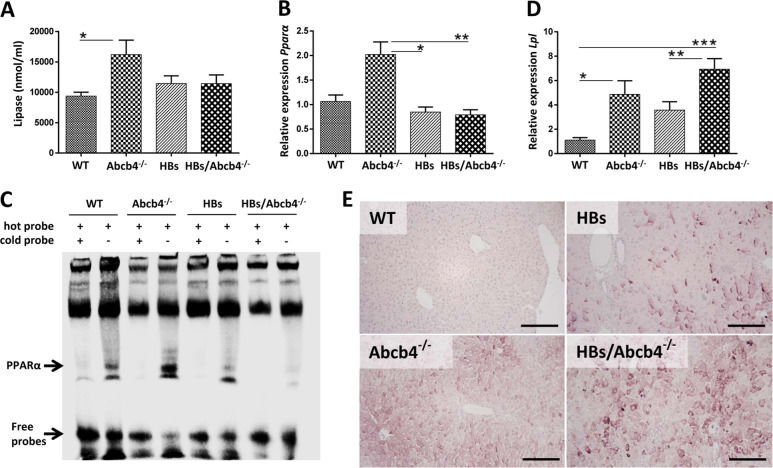
Enhanced lipolysis in HBs/Abcb4^−/−^ mice. **a** Total lipase activity assay was performed using liver lysate prepared as per the protocol given in Cayman lipase assay kit (Cat. No. 700640), *n* = 6 (3♂ + 3♀, age 16–19 weeks). **b**, **c** EMSA demonstrated more efficient binding of PPARα oligonucleotides to nuclear proteins of Abcb4^−/−^ mice in comparison to WT and less efficient in HBs/Abcb4^−/−^ in comparison to HBs. **d** Relative mRNA expression of major genes involved in beta oxidation and lipolysis, PPARα and LPL, in liver of four different groups, relative to WT, normalized against β-actin (PPARα) and GAPDH (LPL) gene. Details of the primers used are listed in Table 1. The results are presented as mean ± SEM, *n* = 7–10 (3–5♂ + 4–5♀, age 16–19 weeks) in each group performed in duplicates. *P < 0.05, **P < 0.01, ***P < 0.001. **e** Representative immunohistochemical analysis of LPL depicts enhanced cytoplasmic expression in Abcb4^−/−^ and HBs/Abcb4^−/−^ in comparison to WT and HBs transgenic male mice. Original image magnification ×200, bar 200 µm.

Increased TAG lipolysis with increased FFA accumulation associated with PPARα activation in Abcb4^−/−^ mice was reported earlier [3]. In order to investigate whether the increase in FFA—as observed in HBs/Abcb4^−/−^ mice (Fig. 1d)—was caused by increased TAG lipolysis, we performed a total lipase activity assay using total liver lysates. We found increased lipase activity in Abcb4^−/−^ mice but no differences between HBs and HBs/Abcb4^−/−^ mice (Fig. 5a).

Since FFAs can activate PPARα, which subsequently regulates the peroxisomal beta oxidation pathway of fatty acids [33], we investigated the PPARα activation in our experimental setup. The transcriptional analysis demonstrated equal expression of *Pparα* in HBs and HBs/Abcb4^−/−^ (Fig. 5b). Interestingly *Pparα* expression was reduced in HBs/Abcb4^−/−^ in comparison to Abcb4^−/−^ (Fig. 5b). EMSA indicated an increased nuclear binding activity of PPARα in Abcb4^−/−^ mice but not in HBs/Abcb4^−/−^-mice (Fig. 5c). PPARα activation subsequently regulates its downstream targets such as LPL and adipose TAG lipase (ATGL) [34]. *Lpl* expression was significantly increased in Abcb4^−/−^ and HBs/Abcb4^−/−^ compared to WT (Fig. 5d). In addition, the LPL protein expression was remarkably increased in HBs/Abcb4^−/−^ compared to HBs mice (Fig. 5e). PPARα activity is, therefore, in line with LPL expression in Abcb4^−/−^-mice but not in HBs/Abcb4^−/−^-mice. Apart from LPL, other lipases such as ATGL or LAL, which are also regulated by PPARα [34], yielded no significant differences in expression (data not shown). Taken together, our results demonstrated an increased hepatic LPL expression in HBs/Abcb4^−/−^ mice.

### *Abcb4* knockout activated AMPK and CREB signaling in HBs/Abcb4^−/−^ mice

**Fig. 6:**
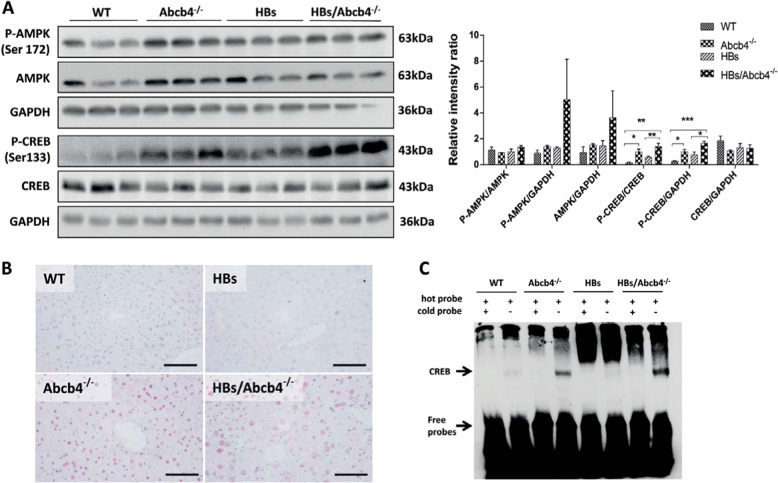
Activation of AMPK and CREB in HBs/Abcb4^−/−^ mice. **a** Western blot analysis using specific antibodies against P-AMPK, AMPK, and P-CREB were performed in male mice. Equal loading was confirmed by GAPDH analysis. **b** Representative immunohistochemical staining using specific antibodies against P-CREB in male mice (original image magnification ×400, bar 200 µm). **c** EMSA demonstrated more efficient binding of CREB oligonucleotides to nuclear proteins of Abcb4^−/−^ and HBs/Abcb4^−/−^ mice in comparison to WT and HBs in male mice.

AMPK is an energy sensor that can induce a cellular cascade for maintaining energy homeostasis [35]. AMPK was activated in Abcb4^−/−^ and HBs/Abcb4^−/−^ compared to WT and HBs (Fig. 6a). CREB and related proteins are downstream targets for AMPK and therefore likely involved in mediating effects of AMPK [36]. Thus, we also investigated the hepatic activation of CREB in Abcb4^−/−^ mice. Immunoblotting demonstrated the increased activation of CREB in Abcb4^−/−^ and HBs/Abcb4^−/−^ compared to wild-type and HBs transgenic mice (Fig. 6a). Similarly, immunohistochemical studies with specific P-CREB antibodies confirmed the above findings (Fig. 6b). Western blot analysis and immunostaining also demonstrated the increased activation of CREB in Abcb4^−/−^, HBs, and HBs/Abcb4^−/−^ compared to wild type in female mice (Supplementary Fig. 3a, b). In addition, EMSA results showed increased nuclear binding of CREB in HBs/Abcb4^−/−^ compared to Abcb4^−/−^, wild-type, and HBs transgenic mice (Fig. 6c). Hence, our data suggest that Abcb4^−/−^ induced cholestatic liver injury might affect hepatic lipid metabolism by the activation of AMPK and CREB signaling in HBs transgenic mice.

### Bile acid treatment induced AMPK–CREB activation in HepG2 cells

**Fig. 7:**
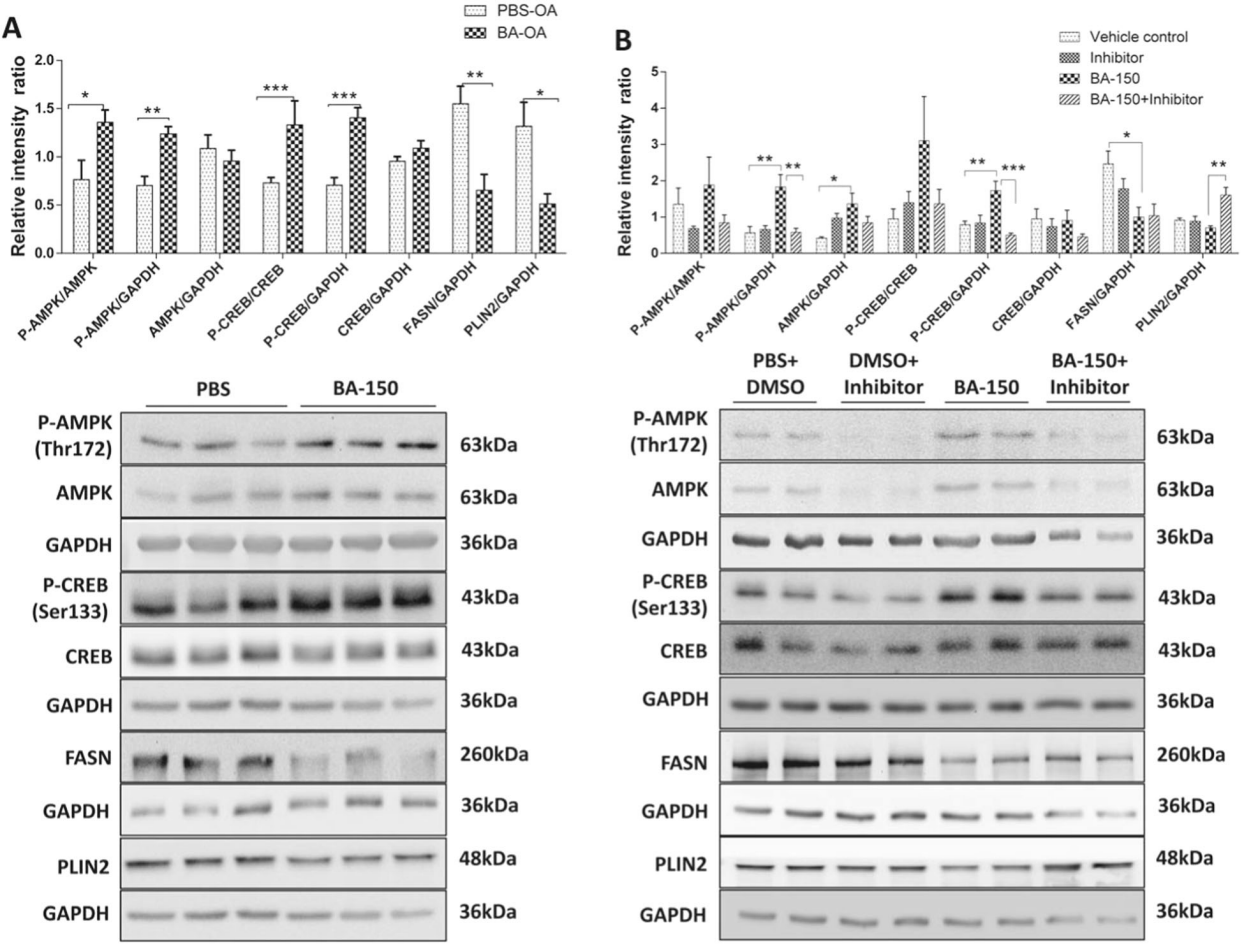
In vitro bile acid treatment induced AMPK and CREB activation in HepG2 cells. **a** Western blot analysis of P-AMPK (α172), AMPK, P-CREB, FAS, and PLIN2 protein expression levels in HepG2 cells pretreated with 250 µM of oleic acid for 12 h followed by treatment with bile acids (cholic-deoxycholate salt) at 150 µM conc. for 24 h. GAPDH was used as a loading control. *n* = 3, each experiment was repeated at least three times. **b** Western blot analysis of P-AMPK (α172), AMPK, P-CREB, FAS, PLIN2 protein expression levels in HepG2 cells pretreated with 250 µM of oleic acid for 12 h followed by treatment with bile acids (cholic-deoxycholate salt) at 150 µM [30, 49] in the presence and absence of dorsomorphin, 10 µM (AMPK inhibitor) conc. for 24 h. GAPDH was used as a loading control. *n* = 3. All experiments were repeated at least three times.

To further prove the mechanistic principle of cholestasis induced disturbances in lipid metabolism in HBs mice, HepG2 cells were used to mimic the situation in vitro. Oleic acid pretreatment of HepG2 cells increased intracellular LDs. The subsequent treatment with BA increased the phosphorylation of AMPK and CREB in HepG2 cells (Fig. 7a). The protein expression of FASN and PLIN2 was reduced in the BA treated group, which was concomitant with the activation of AMPK signaling by bile acid treatment (Fig. 7a). In addition, the LDs associated protein, PLIN2, was reduced remarkably in the treated group compared to vehicle (Fig. 7a). Furthermore, we simulated the in vivo situation by the treatment of HBs overexpressing HepG2 cells with BA. Central aspects like CREB activation and PLIN2 reduction were also demonstrated in this setting (Fig. S4).

To confirm that the effect of bile acid on decreased TAG synthesis and storage was dependent on AMPK–CREB signaling, HepG2 cells preloaded with oleic acid were treated with the specific AMPK inhibitor dorsomorphin in the presence and absence of BA. The activation of AMPK and also CREB by BA was blocked by dorsomorphin (Fig. 7b). Importantly, the downstream targets of AMPK, FASN, and PLIN2, were induced after treatment with the inhibitor (Fig. 7b), suggesting that AMPK activation by BA regulates genes that are involved in lipid synthesis. Our data suggest that bile acid suppresses TAG synthesis and storage in HepG2 cells via activation of the AMPK and CREB signaling pathways.

## Discussion

Cholestasis in NAFLD patients is associated with more pronounced liver injury, inflammation, and disturbances in glucose and lipid metabolism contributing to the progressive course of NAFLD [37]. Clinical studies reported that primary biliary fibrosis and biliary atresia occurred in patients with HBV infection [38, 39]. Similarly, biliary diseases might even be attributed to or caused by HBV infection [39]. We have reported earlier that HBs can enhance cholestatic liver injury, fibrosis, and tumorigenesis in *Abcb4* knockout mice [23]. Therefore, there is an urgent need for medical correction of cholestasis at the earliest form of fatty liver diseases. With regard to chronic HBV infection, concurrent NAFLD represents a “second hit” that can aggravate the disease [6] and the metabolic syndrome is associated with severe fibrosis in CVH [40]. In the current study, we investigated the effect of an *Abcb4* knockout on hepatic lipid metabolism in HBs transgenic mice to understand the relevant biological processes with regard to disease development and progression. Our present study demonstrates reduced hepatic lipogenesis, reduced TAG synthesis, and enhanced lipid catabolism in HBs/Abcb4^−/−^ mice. The activation of AMPK–CREB pathway is suggested to be a major player regulating the changes in hepatic lipid metabolism thereby contributing significantly to the pathogenesis of cholestatic liver injury in HBs mice.

Remarkably, our study revealed a reduction of hepatic TAGs in HBs/Abcb4^−/−^ mice (Fig. 1a–c). Serum TAGs and cholesterol were reduced in HBs/Abcb4^−/−^ mice compared to wild types (Fig. 1g, h). Cholestatic liver disease with toxic accumulation of bile components might disturb many aspects of lipid absorption and metabolism [34]. Furthermore, BA are involved in regulating their own synthesis and enterohepatic circulation, but also TAGs, cholesterol, glucose, and energy homeostasis [41]. Interestingly, we observed increased hepatic FFA levels in Abcb4^−/−^ and HBs/Abcb4^−/−^ mice (Fig. 1d). The induction of hepatic fatty acyl-CoAs and the reduction of TAG esterification and storage has already been described in Abcb4^−/−^ mice [3]. Accordingly, our data also demonstrated a similar effect of Abcb4^−/−^ on enhanced hepatic TAG levels in HBs transgenic mice (Fig. 1a–d).

FFAs have been shown to activate PPARα [42]. The functional nuclear binding of PPARα was increased in our Abcb4^−/−^ mice but nearly no differences were found between HBs and HBs/Abcb4^−/−^ (Fig. 5c). PPARα itself can regulate all sequential steps of TAG catabolism including the regulation of lipases, including LPL [43]. The current data do not provide an explanation for the discrepancy between PPARα and LPL regulation. Nevertheless, PPARα target genes, including lipases, were found to be regulated in HBs/Abcb4^−/−^ mice (Fig. 5d, e). As the liver specific knockout of LPL has no impact on hepatic TG levels [44], it is questionable if the increased expression of LPL, both on transcriptional and translational level in HBs/Abcb4^−/−^ mice in comparison to HBs and WT, might be causal for the reduction of TAGs.

In normal physiological states, FFA serve as a preferential substrate for TAG esterification and storage. Herein, higher levels of FFA, in parallel with low levels of TAGs in Abcb4^−/−^ and HBs/Abcb4^−/−^ indicate either a suppression of the enzyme machinery involved in TAG synthesis or an enhancement of lipolytic activity. The genes and proteins regulating the enzymes involved in TAG synthesis might also contribute to the reduced TAG levels in HBs/Abcb4^−/−^ mice (Fig. 4a–g). TAG biosynthesis from glycerol-3-phosphate is catalyzed by a number of protein isoforms of the glycerol phosphate acyltransferase (GPAT), acylglycerolphosphate acyltransferase (AGPAT), and lipin (phosphatidate phosphatase) families, that appear to catalyze similar biochemical reactions [45]. Notably, AGPAT1 and GPAT1 were significantly reduced in HBs/Abcb4^−/−^ mice (Fig. 4d, e). The protein expression of DGAT1 and the transcriptional level of DGAT2 appeared remarkably decreased in HBs/Abcb4^−/−^ (Fig. 4c, f). The reduced expression of AGPAT1, GPAT1, MGAT1, and DGAT2 suggests that FFAs could not be utilized for TAG synthesis in Abcb4^−/−^ and HBs/Abcb4^−/−^ mice.

Several studies indicated the beneficial effects of lipid storage and loss of TAG storage capacity being critically linked to lipotoxicity, which has been shown to exacerbate liver injury [46]. Therefore, increased FFA levels in parallel with suppression of TAG synthesis and storage along with enhanced lipolysis pathways in HBs/Abcb4^−/−^ mice might also contribute to the acceleration of liver injury [23].

The activation of AMPK inhibits hepatic fatty acid synthesis and promotes fatty acid oxidation via phosphorylation and inactivation of ACC1 [19] and the phosphorylation of specific transcription factors such as SREBP1c by AMPK results in a reduced expression of lipogenic and gluconeogenic enzymes [47]. Our data clearly show an increased activation of AMPK in Abcb4^−/−^ and HBs/Abcb4^−/−^ mice (Fig. 6a). Alongside the increased activation of AMPK, we demonstrate a decrease in genes responsible for lipogenesis, i.e. *Srebp1c*, *Fasn*, *Acc1 Scd1*, *Hmgcs1*, and *Pparγ* in HBs/Abcb4^−/−^ mice (Fig. 2a–f). Also genes which are involved in the uptake and secretion of lipids such as *Cd36*, *ApoE*, and *Pctp* were downregulated in HBs/Abcb4^−/−^ mice. Although *Hmgcs1* expression decreased in HBs/Abcb4^−/−^ mice compared to HBs (Fig. 2e), the hepatic cholesterol level remained constant (Fig. 1e). Nevertheless, serum cholesterol was significantly decreased in HBs/Abcb4^−/−^ mice compared to wild-type mice (Fig. 1h). Cholesterol absorption is dependent on micellar solubilization together with BA and fatty acids [41]. Thus, cholesterol uptake might be affected in HBs/Abcb4^−/−^ mice (Fig. 1h) as elevated plasma bile acid levels are a characteristic hallmark in Abcb4^−/−^ mice [48] as well as in HBs/Abcb4^−/−^ mice [23].

Previous studies reported that the AMPK-mediated phosphorylation of the LD-associated protein PLIN2 is essential for subsequent selective degradation and triggering of lipolysis [22], suggesting an interplay between AMPK and PLIN2 in regulating intracellular TAG storage. PLIN2, being the major LD-associated protein, protects and stabilizes LDs, provides a “shielding effect” and modulates lipase’s accessibility to TAGs [22]. Furthermore, the reduction of PLIN2 expression by antisense oligonucleotide treatment led to decreased hepatic lipid accumulation [49]. However, the association of Abcb4 mediated reduction of TAGs and PLIN2 has not been reported so far. Here our study showed a reduction of PLIN2 both in vivo and in vitro (Figs. 3a–c and 7a, b) suggesting a role of cholestasis mediated regulation on PLIN2 expression.

Previous reports suggested that CREB could be induced by AMPK [50]. These observations, along with scanning of peptide sequences for AMPK recognition motifs, led to the hypothesis that CREB may influence lipid homeostasis together with AMPK [36]. Notably, CREB was activated in HBs/Abcb4^−/−^ in comparison to Abcb4^−/−^ and HBs mice (Fig. 6a, b). In addition to its role in promoting fatty acid breakdown, CREB also appears to block the expression of lipogenic pathways [20]. Intriguingly, our study also shows a decreased expression of PPARγ in HBs/Abcb4^−/−^ mice (Fig. 2f). Mice deficient in CREB activity have a fatty liver phenotype and display an elevated expression of the nuclear hormone receptor PPAR-γ, a key regulator of lipogenic genes [20]. Similarly, our cell culture models of cholestasis demonstrated increased activation of AMPK and CREB along with downregulation of FASN and PLIN2 in HepG2 cells, emphasizing the possible role of bile acid in lipid metabolism during cholestatic liver injury (Fig. 7a, b). Finally, AMPK inhibition reversed this effect, which underlines a mechanistic association.

In summary, the hepatic lipid metabolism in HBs mice was altered by concomitant cholestasis induced via *Abcb4* transporter knockout. AMPK and CREB signaling could mediate this process. The results of the current study may trigger the development of novel therapeutic strategies as NASH is a crucial factor able to aggravate chronic liver disease in HBV infected patients [6]. In conclusion, a pharmacological modulation of AMPK and CREB signaling might be a promising therapeutic concept for the treatment of fatty liver diseases.

## Supplementary information

Supplemental
