# Supplementary material for: Cholestasis impairs hepatic lipid storage via AMPK and CREB signaling in hepatitis B virus surface protein transgenic mice
Source: Lab Invest. 2020 Jul 1;100(11):1411–24. doi: 10.1038/s41374-020-0457-9 (PMC7572243; doi:10.1038/s41374-020-0457-9)
Supplement: Supplementary file 1 — Supplemental [file 41374_2020_457_MOESM1_ESM.pdf]

SUPPLEMENTAL INFORMATION:

Cholestasis impairs hepatic lipid storage via AMPK and CREB signaling in hepatitis B virus surface protein transgenic mice

Running Title: Cholestasis reduced steatosis

Karuna Irungbam[1], Martin Roderfeld[1], Hannah Glimm[1], Felix Hempel[1], Franziska Schneider[1], Laura Hehr[1], Dieter Glebe[2], Yuri Churin[1], Gertrud Morlock[3], Imanuel Yüce[3], Elke Roeb[1,*]

[1]Department of Gastroenterology, Justus Liebig University Giessen, Giessen, Germany
[2]Institute of Medical Virology, National Reference Centre for Hepatitis B and D Viruses, Justus Liebig University, Giessen, Germany
[3]Institute of Nutritional Science, Chair of Food Science, and TransMIT Center for Effect-Directed Analysis, Justus Liebig University Giessen, Giessen, Germany

Supplemental Fig. S1

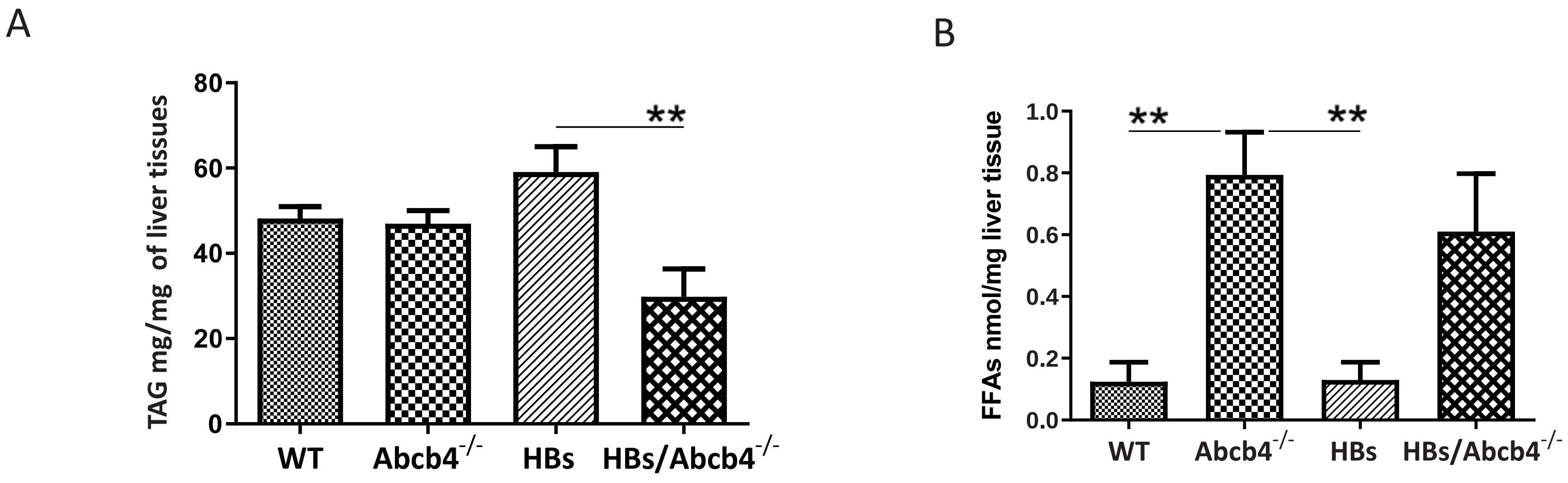

Fig S1. Abcb4$^{-/-}$ induced cholestasis mediates reduction of liver TAGs and increased FFAs in HBs mice.

(A) Quantification of TAGs from liver lysates using enzymatic based assays (Cayman, cat no.10010303) showed a reduction in TAGs in HBs/Abcb4$^{-/-}$ mice; n= 5-6 (2-3♂+3♀, age 16-19 weeks). **(B)** FFAs quantification was performed in liver lysates using enzymatic methods (Promokine, cat no. PK-CA577-K612). FFAs were significantly increased in Abcb4$^{-/-}$ to WT and HBs and tendentially increased in HBs/Abcb4$^{-/-}$ in comparison to HBs (n=0.057), n= 6. P**<0.01.

Supplemental Fig. S1

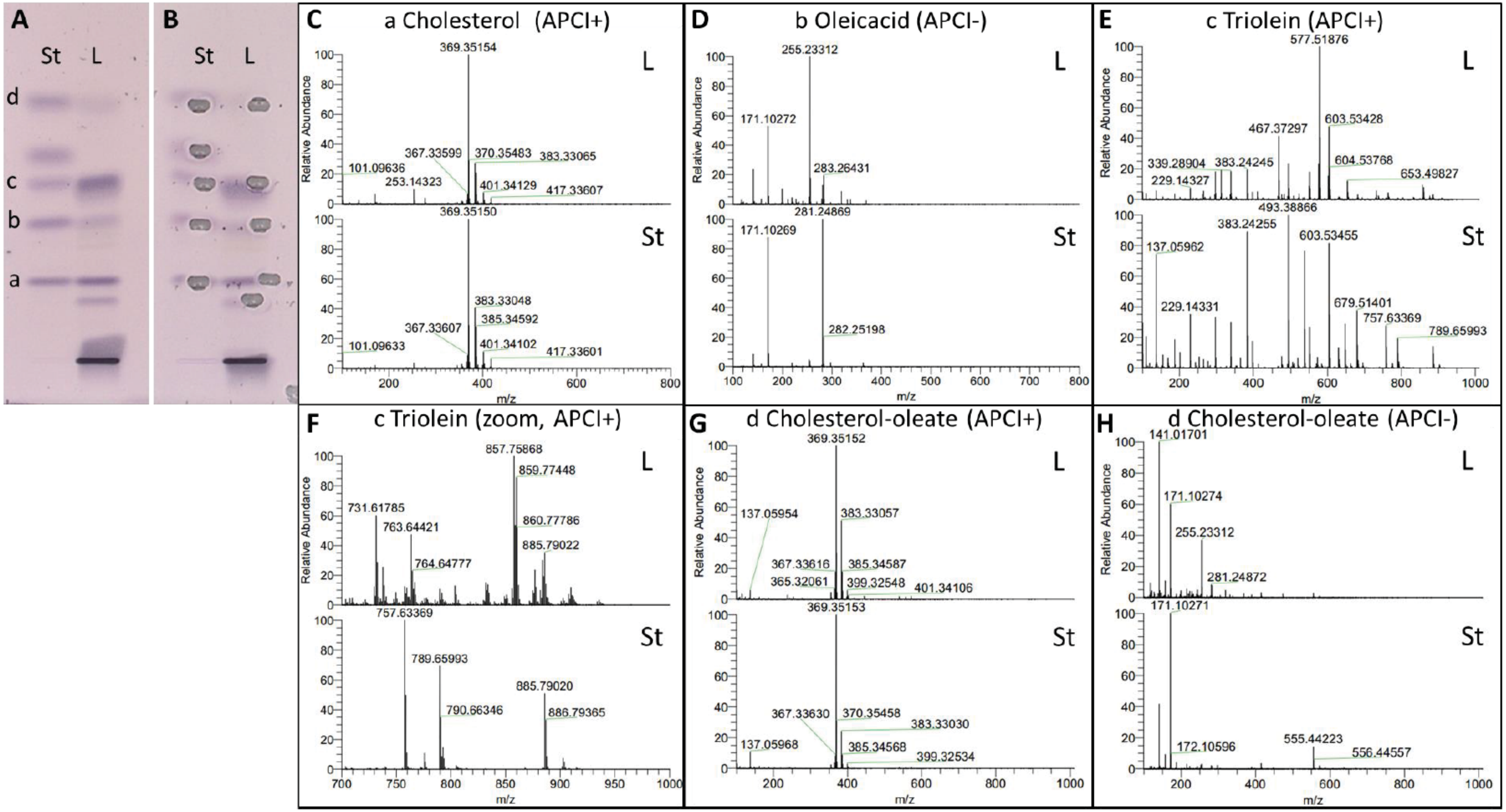

Figure S1 (C) HPTLC plates loaded with lipids (A) and selected zones online eluted (B) via TLC-MS Interface 2 into the HRMS, HPTLC-HRMS mass spectra (C-H) of four different lipids (band a-d) from the liver samples (L) and lipid standards (St).

Supplemental Fig. S2

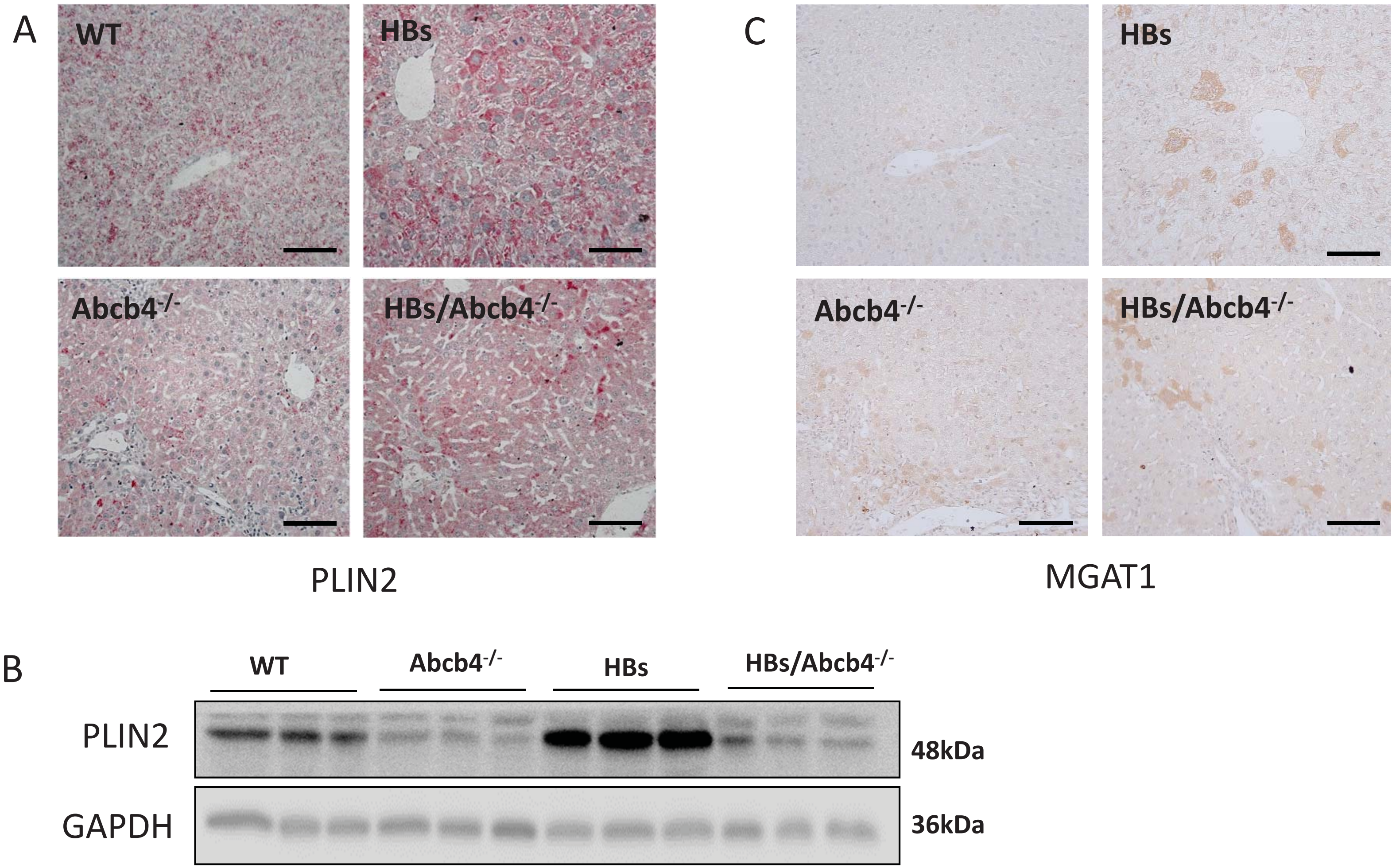

Fig S2. PLIN2 and MGAT1 expression in female mice.
(A) Representative immunohistochemical analysis of paraffin-embedded liver sections from 16 weeks old female mice was performed using anti-PLIN2 antibodies. PLIN2 is stained red. Original magnification x 200, bars 100 µm. **(B)** Representative western blot analysis of lysates from the liver of 16 weeks old female mice. Equal protein loading was confirmed using anti-GAPDH antibodies. (C) Representative immunohistochemical analysis of paraffin-embedded liver sections from 16 weeks old female mice was performed using anti-MGAT1 antibodies. Original magnification x 200, bars 100 µm.

Supplemental Fig. S3

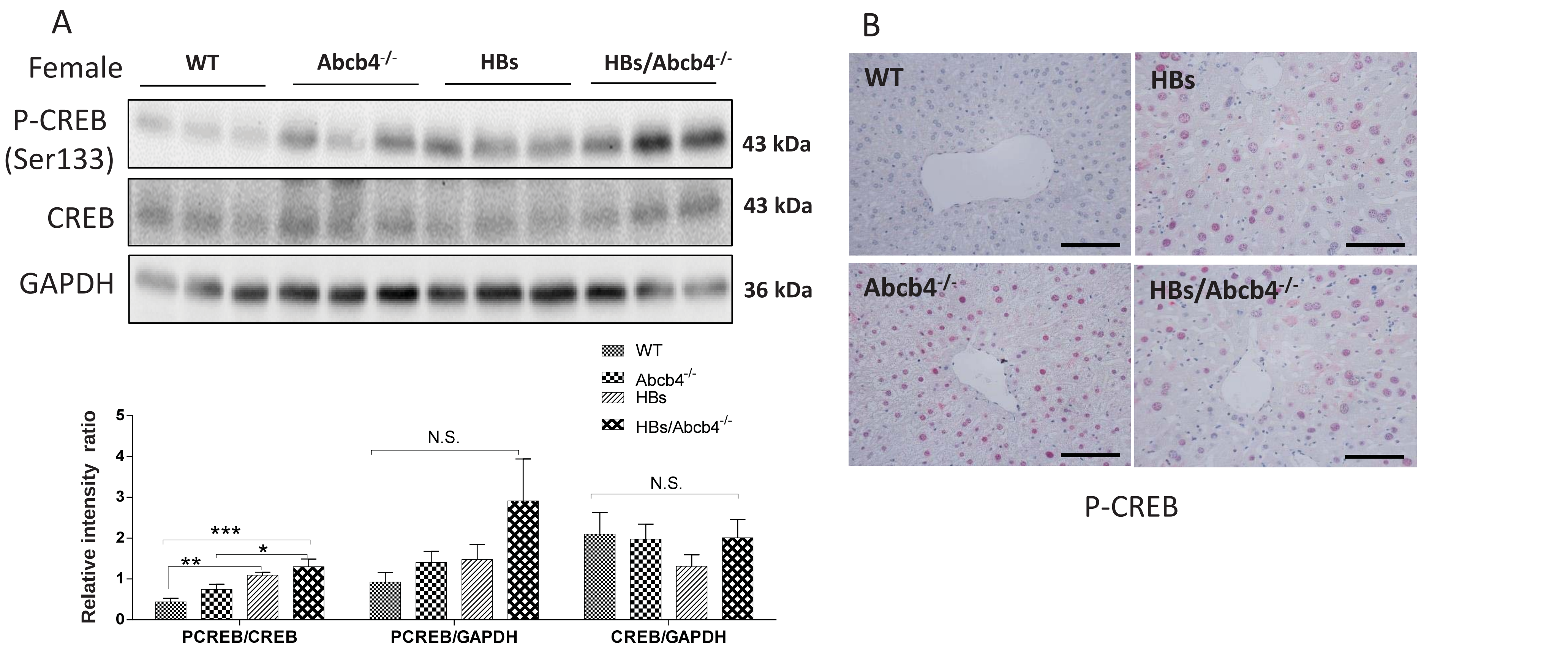

Fig S3. CREB activation in female mice.
(A) Representative western blot analysis of lysates from the liver of 16 weeks old female mice was performed using specific anti-phospho-CREB antibodies. Equal protein loading was confirmed using CREB and anti-GAPDH antibodies. **(B)** Representative immunohistochemical analysis of paraffin-embedded liver sections from 16 weeks old mice was performed using anti-P-CREB antibodies. Original magnification x 400, bars 100 µm.

Supplemental Fig. S4

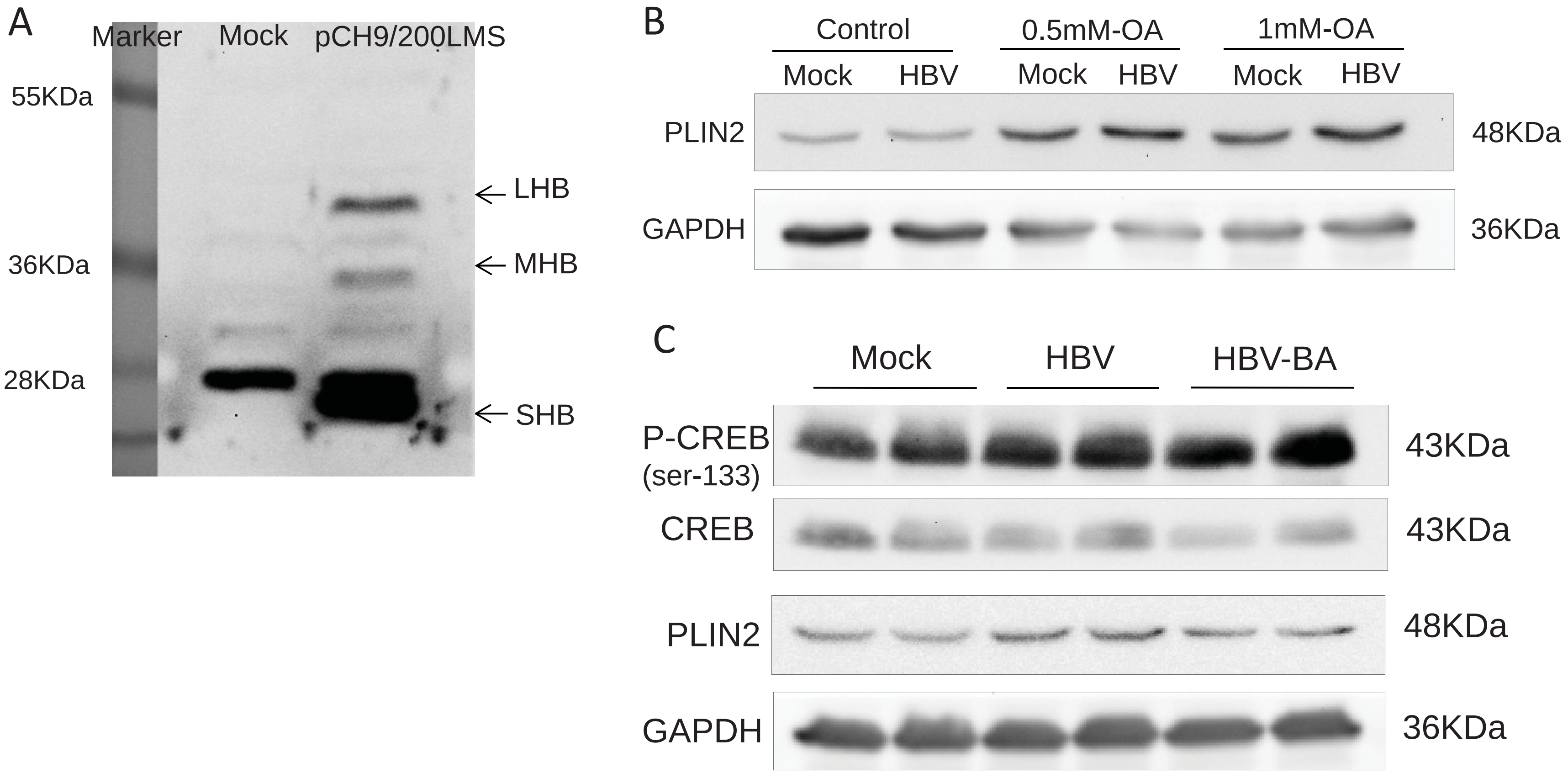

Fig S4. HBs induced CREB activation and PLIN2 expression is modulated by bile acids.
(A) pCH9/200LMS transfected HepG2 cells expressed normal composition of the three HBV surface proteins LHB, MHB and SHB (large, middle and small HBV surface proteins). The plasmid pCH9/200LMS is a replication-defective variant of plasmid pCH9/3091[35] and encodes the HBV surface proteins under their natural promoters. HepG2 cells with 80% confluence were transfected with and without the plasmid pCH9/200LMS for 48h, followed by treatment with oleic acid for 12h. After that treated with bile acid in serum free DMEM for the next 24h. **(B)** Western blot analysis demonstrated HBs potentiated PLIN2 expression after OA treatment in HepG2 cells. **(C)** Phosphorylation of CREB is induced by HBs expression and potentiated by BA. HBs induced PLIN2 expression is repressed by BA. Representative blots are presented.
